# The Vaginal Microbiome–Mitochondria Axis: Implications for Genital Tract Infections

**DOI:** 10.3390/cells15201835

**Published:** 2026-10-09

**Authors:** Lorenzo Agoni, Eva Pericolini, Stefano Salvatore, Massimo Candiani, Francesco De Seta

**Affiliations:** 1 Unit of Obstetrics and Gynecology, Fondazione Poliambulanza Istituto Ospedaliero, 25124 Brescia, Italy; lorenzo.agoni@poliambulanza.it; 2 Department of Surgical, Medical, Dental and Morphological Sciences with Interest in Transplant, Oncological and Regenerative Medicine, University of Modena and Reggio Emilia, 41124 Modena, Italy; eva.pericolini@unimore.it; 3 Department of Obstetrics and Gynecology, IRCCS San Raffaele Scientific Institute, 20132 Milan, Italy; salvatore.stefano@hsr.it (S.S.); candiani.massimo@hsr.it (M.C.); 4 Sbarro Institute for Cancer Research and Molecular Medicine and Center of Biotechnology, College of Science and Technology, Temple University, Philadelphia, PA 19122, USA

**Keywords:** vaginal microbiome, mitochondria, vaginal dysbiosis, host–microbe interactions, microbial metabolites, mitochondrial dysfunction, recurrent vaginal infections

## Abstract

**Highlights:**

**What are the main findings?**

- Microbial metabolites and virulence factors relevant to vaginal health and disease can modulate mitochondrial bioenergetics, redox signaling, dynamics, and quality control, providing a mechanistic basis for microbiome–mitochondria communication.
- Mitochondrial responses may translate microbial signals into distinct epithelial and immune outcomes, supporting the concept of a functional vaginal microbiome–mitochondria axis.

**What are the implications of the main findings?**

- Failure to restore mitochondrial homeostasis after microbial control may contribute to an incomplete microbial ecosystem–host reset and represents a testable mechanism potentially involved in recurrence.
- The proposed axis expands therapeutic thinking beyond microbial eradication toward restoration of microbial ecosystem–host function through direct or microbiota-mediated modulation of mitochondrial homeostasis.

**Abstract:**

The vaginal microbiome influences epithelial and immune homeostasis through microbial metabolites, virulence factors, and other molecular signals, yet intracellular mechanisms translating microbial activity into host responses remain largely unexplored. Here, we propose the vaginal microbiome–mitochondria axis as a functional framework linking microbial activity to host-cell metabolism, redox signaling, immunity, and cell fate. We integrated evidence from vaginal microbiology, mitochondrial biology, mucosal immunology, and immunometabolism, distinguishing direct observations in the female genital tract from mechanistic evidence obtained in other host–microbe systems. Available evidence indicates that microbial products relevant to vaginal homeostasis and dysbiosis can modulate mitochondrial bioenergetics, reactive oxygen species generation and redox signaling, membrane potential, dynamics, and quality control pathways, while mitochondrial responses can influence epithelial integrity and antimicrobial and inflammatory functions. We further propose that the balance between mitochondrial adaptation and dysfunction may contribute to resolution or persistence of vaginal disorders and that incomplete mitochondrial recovery could contribute to an incomplete microbial ecosystem–host reset and recurrence. Although direct vaginal evidence remains limited, this framework identifies mitochondria as a potentially important functional interface between microbial ecology and host response and generates testable hypotheses for investigating whether mitochondrial homeostasis contributes to durable resolution of vaginal dysbiosis and infection.

## 1. Introduction

The vaginal microbiome is a dynamic microbial ecosystem that contributes to the maintenance of female genital tract homeostasis. In reproductive-age women, vaginal health is generally associated with low-diversity communities dominated by *Lactobacillus* species, whereas changes in microbial composition and function characterize conditions such as bacterial vaginosis (BV), aerobic vaginitis (AV), and vulvovaginal candidiasis (VVC). These conditions differ substantially not only in their microbial features but also in the epithelial and immune responses they elicit. Microbial composition alone therefore cannot fully explain the biological behavior of the vaginal ecosystem or the heterogeneous host responses associated with vaginal dysbiosis and infection.

Increasing attention has consequently shifted from microbial taxonomy toward microbial function and microbe–host interactions. Vaginal microorganisms generate a complex repertoire of metabolites, virulence factors, and other molecular signals capable of modifying epithelial and immune cell behavior. The biological consequences of these signals depend not only on their production, but also on how they are sensed and processed by host cells in the vaginal environment. The vaginal mucosa should therefore be regarded not as a passive substrate for microbial colonization or infection, but as an active component of host defense that interacts with resident and pathogenic microorganisms and contributes to shaping the local environment. Identifying the intracellular mechanisms that translate microbial activity into metabolic, inflammatory, and antimicrobial responses is therefore essential for understanding how different vaginal microbial states produce different biological outcomes.

Mitochondria are well positioned to participate in this process. Beyond ATP production, they regulate cellular metabolism, redox signaling, innate immunity, inflammatory responses, and cell fate. Their bacterial ancestry has endowed mitochondria with molecular features that engage innate immune and cellular stress pathways, providing an evolutionary link between microbial sensing and host-cell responses. In several host–microbe systems, particularly at mucosal surfaces, microbial metabolites and virulence factors can modify mitochondrial respiration, reactive oxygen species production, membrane potential, dynamics, and quality-control pathways, while mitochondrial activity can in turn influence epithelial barrier function and immune responses. Direct evidence in the female genital tract remains limited, but emerging observations indicate that vaginal microorganisms and their products can also interact with mitochondrial pathways in epithelial and immune cells.

In this review, we propose the vaginal microbiome–mitochondria axis as a functional framework linking microbial activity to host-cell responses in the vaginal environment. Rather than proposing a previously unrecognized role for mitochondria in host–microbe interactions, the novelty of this framework lies in integrating established mitochondrial functions and mechanistic observations from other host–microbe systems with the emerging evidence available in the female genital tract, and in applying this integrated perspective specifically to the vaginal ecosystem. We examine how microbial metabolites and virulence factors may influence mitochondrial function, how mitochondria may translate these signals into epithelial and immune responses, and how the balance between mitochondrial adaptation and dysfunction could contribute to resolution or persistence of vaginal disorders. We further explore the hypothesis that incomplete mitochondrial recovery may participate in failure of microbial ecosystem–host reset and recurrence and consider whether the axis could ultimately be targeted through direct modulation of mitochondrial homeostasis or indirectly through microbial-derived interventions. Because much of the mechanistic evidence currently derives from non-vaginal experimental systems, the proposed axis is presented not as an established pathogenic pathway, but as a hypothesis-generating model from which specific and experimentally testable predictions can be derived.

## 2. Literature Search and Conceptual Approach

This narrative review integrates evidence from vaginal microbiology, mitochondrial biology, mucosal immunology, and immunometabolism to develop a conceptual framework for microbiome–mitochondria interactions in the female genital tract. Relevant literature was identified primarily through PubMed searches and examination of the reference lists of pertinent original studies and reviews. Searches were iterative and focused on combinations of concepts related to the vaginal microbiome and dysbiosis, mitochondrial function, microbial metabolites and virulence factors, redox signaling, epithelial and immune responses, and neutrophil function. Searches were progressively refined to explore specific microbial products and mitochondrial pathways identified during the literature review. Literature searches were updated through September 2026.

Given the conceptual scope of the review, study selection was based on biological relevance to the proposed framework rather than on systematic review methodology. Emphasis was placed on studies providing mechanistic information on microbe–host interactions, microbial metabolites, mitochondrial responses, redox signaling, epithelial immunity, neutrophil function, and NET formation.

Evidence was interpreted according to its biological context. Direct experimental or clinical findings from the female genital tract were distinguished from mechanistic observations obtained in other tissues or host–microbe systems. The latter were used to identify biologically plausible connections and generate hypotheses, rather than as evidence that the same mechanisms have been demonstrated in the vaginal environment. Accordingly, this review is intended as a critical and hypothesis-generating synthesis and not as a systematic or exhaustive assessment of the literature.

## 3. From Vaginal Microbial Composition to Host Response

The vaginal microbiota has been extensively characterized and categorized according to its taxonomic composition, with low-diversity, *Lactobacillus*-dominated communities generally associated with vaginal health [1]. However, taxonomic composition alone provides limited information on how a given microbial community interacts with the host and elicits a local biological response [1,2].

The vaginal mucosa is an active component of this interaction rather than simply the anatomical substrate on which microbial communities reside. Vaginal epithelial cells contribute to microbial sensing, antimicrobial defense, and regulation of local inflammatory responses [3], while epithelial integrity, turnover, and metabolic activity help shape the physicochemical and nutritional environment encountered by microorganisms [3,4]. The relationship is therefore reciprocal: microbial communities influence mucosal function, while mucosal activity contributes to determining the ecological conditions in which resident microorganisms and potential pathogens persist [3,4].

Bacterial vaginosis is characterized by the loss of *Lactobacillus* dominance, increased microbial diversity, and the development of a polymicrobial biofilm on the vaginal epithelium [5]. Despite these marked ecological changes, BV is not typically associated with prominent vaginal neutrophil infiltration [5,6]. This limited neutrophil recruitment does not imply the absence of a mucosal immune response. BV–associated bacteria can induce epithelial cytokine production while, among other mechanisms, bacterial enzymes and metabolites can interfere with IL-8 signaling and leukocyte chemotaxis, and *Gardnerella vaginalis*-derived vaginolysin can directly lyse neutrophils [5,6]. Thus, the host response in BV cannot be described simply as activated or suppressed, as different components of the mucosal immune response may be differentially affected.

Aerobic vaginitis provides a different model of microbe–host interaction. In AV, loss of lactobacillary dominance is associated with a marked inflammatory response, characterized by increased numbers of leukocytes, including toxic leukocytes, and increased levels of pro-inflammatory cytokines such as IL-1β and IL-6 [7,8]. However, the inflammatory response associated with AV does not appear to depend solely on the presence of bacterial species commonly associated with this condition. Experimental evidence suggests that this response may also depend on strain-specific properties. In vaginal epithelial cells, for example, a pathogenic strain of *Enterococcus faecalis* induced IL-8 production, whereas a strain isolated from the vaginal microbiota of a healthy woman did not under the same experimental conditions, indicating that taxonomic identity alone does not predict the inflammatory potential of a vaginal isolate [9].

Vulvovaginal candidiasis further illustrates that the intensity of an inflammatory response does not necessarily correspond to its effectiveness in controlling the microorganism. During symptomatic *Candida albicans* infection, hyphal growth and the release of candidalysin induce epithelial inflammatory signaling and promote neutrophil recruitment [10,11,12]. Nevertheless, abundant neutrophil recruitment does not necessarily result in effective fungal clearance. Vaginal heparan sulfate, for example, can interfere with neutrophil recognition and killing of *C. albicans*, providing a mechanism by which an intense inflammatory response may coexist with impaired antifungal activity [13,14]. The magnitude of the inflammatory response also varies among *Candida* species. In experimental VVC, several non-*albicans Candida* species induced less neutrophil recruitment and mucosal damage than *C. albicans*, despite comparable early vaginal colonization [15]. Thus, the host response to *Candida* depends not only on fungal burden but also on species-specific virulence properties, while the magnitude of neutrophil recruitment does not necessarily correspond to effective fungal clearance.

Taken together, BV, AV and VVC illustrate that the nature and magnitude of the vaginal host response cannot be inferred from microbial composition alone but also depend on the specific molecular and metabolic signals generated during microbe–host interactions and on how host cells respond to these signals. Understanding how microbial signals are processed by host cells may therefore help explain the different patterns of epithelial and immune responses observed across vaginal conditions. In the following sections, we propose that mitochondria may represent an intracellular interface through which microbial signals are integrated into host cellular responses.

## 4. Mitochondria at the Host–Microbe Interface

Mitochondria are central regulators of cellular metabolism, coupling substrate oxidation to ATP production and biosynthetic processes [16]. Beyond bioenergetics, they contribute to redox homeostasis, innate immune signaling, and cell-fate regulation through mitochondrial metabolites, mitochondrial reactive oxygen species (mtROS), nucleic acids, and mitochondria-associated signaling pathways [17,18,19].

Mitochondria originated from an ancestral bacterial endosymbiont and retain several molecular features of their bacterial ancestry. These include a circular genome, bacterial-like protein synthesis, N-formylated peptides, and cardiolipin-rich membranes. Under physiological conditions, these components are largely compartmentalized within mitochondria and do not trigger an immune response. However, mitochondrial stress or damage can expose or release mitochondrial molecules into the cytosol or extracellular environment, where they can be recognized by innate immune pathways that also participate in microbial sensing [20]. This evolutionary legacy therefore provides a biological link between mitochondrial function and mechanisms involved in the cellular response to microorganisms.

Evidence from other host–microbe systems supports bidirectional communication between microbial communities and mitochondria [21,22]. This has been particularly investigated in the intestinal mucosa, where microbial metabolites can modify mitochondrial function in epithelial and immune cells [23,24,25]. A well-characterized example is microbiota-derived butyrate, which promotes mitochondrial respiration and oxygen consumption in colonocytes, thereby reducing epithelial oxygen availability and helping maintain the hypoxic environment that favors obligate anaerobes [24,25]. Conversely, impaired epithelial mitochondrial metabolism can increase local oxygen availability and alter microbial ecology [24,25]. This reciprocal relationship provides a mechanistic basis for considering mitochondria not only as targets of microbial activity, but also as contributors to the environment in which microbial communities reside.

In the vaginal environment, direct evidence linking microbial activity to mitochondrial responses remains limited, but it is beginning to emerge. A direct experimental example has been provided for *Candida albicans* in vaginal epithelial cells. In a reconstituted vaginal epithelium model, *C. albicans* induced a rapid increase in mtROS, with candidalysin playing a major role in mitochondrial activation [12]. Importantly, the pharmacological reduction in mtROS resulted in increased fungal burden, suggesting that the mitochondrial response was not merely a consequence of epithelial damage but contributed to the host response to the microorganism [12]. These findings provide proof of principle that a vaginal microorganism can generate signals capable of modifying mitochondrial activity in host epithelial cells and that the resulting mitochondrial response can influence the interaction between host and microorganism.

**Figure 1:**
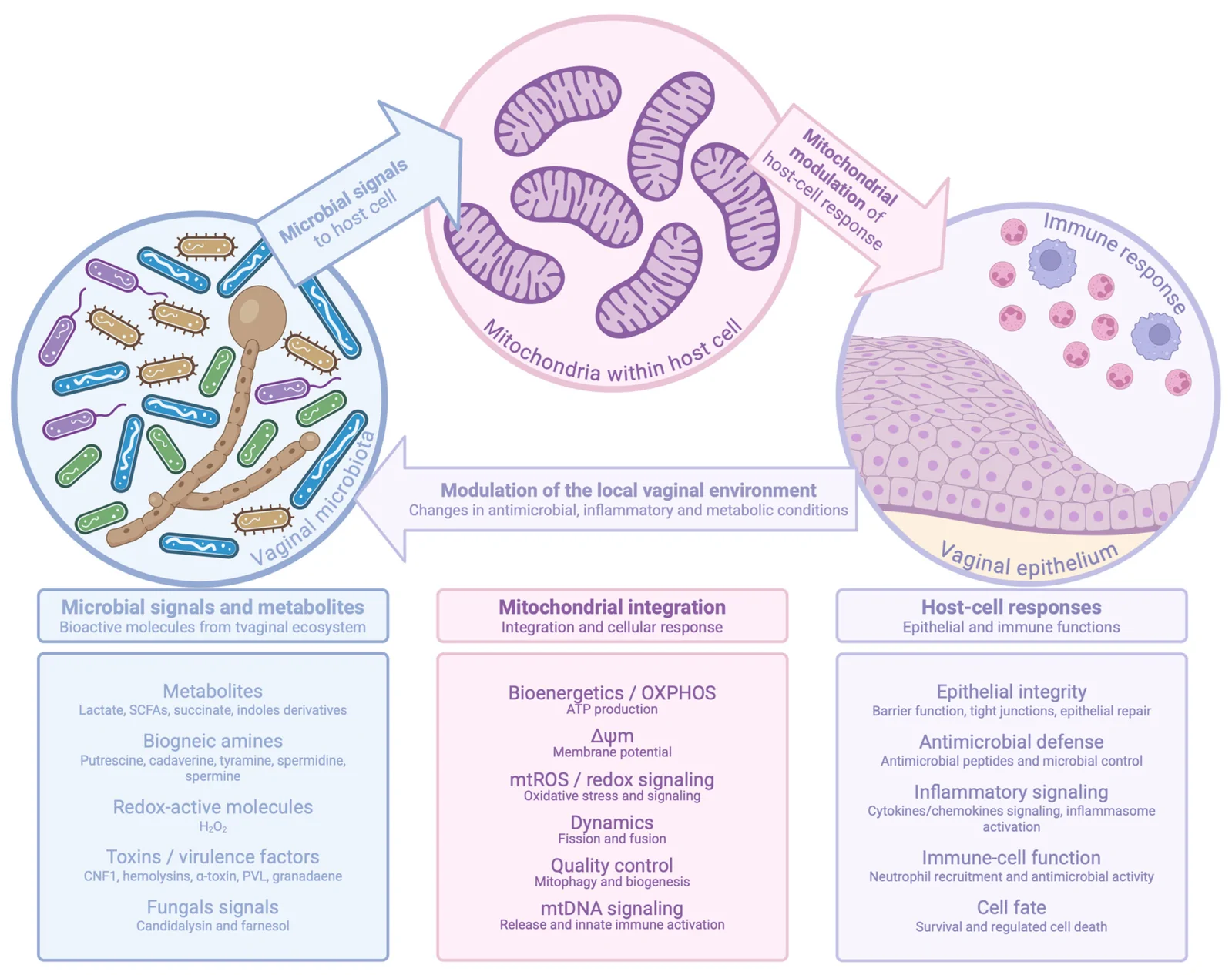
Proposed mechanisms linking vaginal microbial signals to mitochondrial function and host-cell responses. Microbial metabolites, biogenic amines, redox-active molecules, and bacterial and fungal virulence-associated signals generated within the vaginal ecosystem may influence mitochondrial function in host cells. Mitochondria integrate these inputs through changes in bioenergetics and oxidative phosphorylation (OXPHOS), mitochondrial membrane potential (ΔΨm), mitochondrial reactive oxygen species (mtROS) and redox signaling, mitochondrial dynamics, quality-control pathways, and mitochondrial DNA (mtDNA) signaling. These responses may modulate epithelial integrity, antimicrobial defense, inflammatory signaling, immune-cell function, and cell fate. Host-cell responses can, in turn, modify the local vaginal environment through changes in antimicrobial, inflammatory, and metabolic conditions, potentially influencing microbial community structure and activity. The figure therefore illustrates the proposed bidirectional nature of the vaginal microbiome–mitochondria axis. The depicted mechanisms integrate direct vaginal evidence with mechanistic findings from other experimental systems and should not be interpreted as having all been demonstrated in the vaginal environment. OXPHOS, oxidative phosphorylation; ΔΨm, mitochondrial membrane potential; mtROS, mitochondrial reactive oxygen species; mtDNA, mitochondrial DNA; SCFAs, short-chain fatty acids; CNF1, cytotoxic necrotizing factor 1; PVL, Panton–Valentine leukocidin. Created in https://BioRender.com.

Based on these observations, we propose that a similar functional relationship may operate within the vaginal ecosystem. We define the vaginal microbiome–mitochondria axis as a functional communication network through which metabolic and molecular signals generated by the vaginal microbial ecosystem may modulate mitochondrial function in mucosal epithelial and immune cells, thereby influencing host responses that can, in turn, modify the local vaginal environment. Within this framework, the vaginal mucosa represents an active biological interface in which epithelial and immune responses contribute to microbial control while simultaneously shaping the local environment encountered by resident and pathogenic microorganisms. In this model, mitochondria are not considered passive targets of microbial activity, but intracellular integrators capable of converting microbial and microenvironmental signals into changes in cellular metabolism, redox signaling, immune function, and cell fate [26,27]. The resulting host response may, in turn, modify the vaginal ecosystem, establishing a potentially bidirectional interaction between microbial activity and mitochondrial function (Figure 1). The causal direction of these interactions remains unresolved in the vaginal environment. Available vaginal evidence supports the capacity of microbial signals to induce mitochondrial responses, as demonstrated experimentally for *C. albicans* and mtROS [12]. Whether mitochondrial alterations subsequently contribute to changes in the vaginal microbial ecosystem, rather than representing only downstream responses to microbial activity, has not been established. Mitochondrial alterations may therefore represent consequences of microbial perturbation, functional mediators of the host response, or, if persistent or dysregulated, potential contributors to conditions that influence microbial persistence or recurrence. The bidirectional relationship proposed here should consequently be regarded as a testable hypothesis rather than an established causal mechanism. Distinguishing among these possibilities will require experimental approaches specifically designed to establish temporal sequence and causality in vaginal models.

The proposed axis integrates evidence at different levels of biological support. Some components are supported by direct observations in vaginal models [12], whereas others derive from mechanistic studies performed in different mucosal tissues or host–microbe systems [28,29]. These latter findings provide biological plausibility and identify mechanisms that may operate in the vaginal environment, but they should not be interpreted as evidence that the same interactions have already been demonstrated in this setting. Accordingly, the vaginal microbiome–mitochondria axis should be regarded as a hypothesis-generating framework that connects established observations with biologically plausible mechanisms and identifies specific interactions that remain to be experimentally tested in the female genital tract, following the broader conceptual use of axis-based frameworks to integrate interconnected microbial ecosystems and host physiology [30].

## 5. Microbial Metabolism as Mitochondrial Information

Taxonomic composition describes the structure of a microbial community but does not fully capture its functional activity. Microorganisms continuously transform available substrates and generate metabolites and other molecular products that modify the biochemical environment in which they reside. In the vagina, changes in microbial community structure are accompanied by substantial changes in the local metabolic profile [31,32]. These molecules are not merely markers of microbial composition but can interact with epithelial and immune cells and thereby contribute to the functional relationship between the microbiota and the host [32,33]. From this perspective, the metabolic output of the vaginal ecosystem represents a potential source of biological information through which changes in microbial activity may be communicated to host cells.

Lactic acid provides a particularly relevant example of how microbial metabolism can generate biologically active signals within the vaginal environment. In reproductive-age women with a *Lactobacillus*-dominated microbiota, lactic acid is produced predominantly through bacterial fermentation of carbohydrates derived from the vaginal environment and represents the major determinant of vaginal acidity [34,35]. Its biological activity, however, extends beyond acidification. In cervicovaginal epithelial cells, lactic acid can modulate immune signaling, increasing IL-1 receptor antagonist (IL-1RA) production while reducing the production of several pro-inflammatory mediators in response to inflammatory stimuli [36]. Importantly, these effects depend on the physicochemical context: at physiological vaginal acidity, the protonated form of lactic acid appears to account for much of its immunomodulatory activity [35,36]. Because lactic acid has a pKa of approximately 3.86, the acidic conditions characteristic of a *Lactobacillus*-dominated vaginal environment increase the proportion present in the protonated, uncharged form, which can cross cell membranes more readily than the lactate anion [35]. Thus, lactic acid illustrates how a metabolic product generated by the vaginal microbiota can simultaneously modify the extracellular environment and directly influence host-cell function.

Following cellular uptake, lactate can participate in metabolic and signaling pathways closely connected to mitochondrial function. Evidence from several tissues, including skeletal muscle, brain, and epithelial cells, shows that lactate can link glycolytic and oxidative metabolism, influence cellular redox balance, and modify mitochondrial respiration and bioenergetic adaptation [37,38]. In alveolar epithelial cells, for example, lactate utilization has been directly linked to mitochondrial ATP production and increased oxidative metabolism, demonstrating that epithelial cells can use lactate to modulate their bioenergetic state [39]. Beyond its role as a metabolic substrate, lactate can also function as a signaling molecule and influence cellular responses independently of its contribution to energy metabolism [38]. Together, these observations provide a mechanistic basis for hypothesizing that microbiota-derived lactic acid/lactate in the vaginal environment may influence host-cell physiology both through extracellular effects associated with vaginal acidification and through modulation of intracellular metabolic pathways connected to mitochondrial function. Whether this occurs in vaginal epithelial or immune cells, and to what extent it contributes to the biological effects attributed to lactic acid in the vaginal environment, remains to be established.

Beyond lactic acid and lactate, the transition from a *Lactobacillus*-dominated ecosystem to more diverse microbial communities is accompanied by a broader metabolic reconfiguration, potentially exposing epithelial and immune cells to a different combination of microbial products [31,40,41]. Experimental evidence from different biological systems indicates that several of these products, potentially relevant to vaginal homeostasis, bacterial vaginosis, aerobic vaginitis, or vulvovaginal candidiasis, can influence mitochondrial metabolism, redox signaling, bioenergetics, and dynamics, although these effects have rarely been investigated directly in vaginal host cells (Table 1). The molecular output of the vaginal microbiota may therefore represent not simply a biochemical consequence of microbial community composition, but a functionally distinct interface through which different microbial ecosystems generate different signals to the host. Which of these signals reach or modify mitochondrial function in vaginal cells, however, remains largely unexplored.

## 6. Mitochondria as Translators of Vaginal Host Response

The biological significance of microbiota–mitochondria communication depends ultimately on whether mitochondrial responses to microbial signals modify the behavior of host cells. A microbial metabolite or virulence factor may alter mitochondrial function, but this alteration becomes relevant to the microbe–host interaction only when it changes how epithelial or immune cells respond to the microbial stimulus. In epithelial cells, mitochondrial responses to microbial signals may influence cellular metabolism, barrier function, and epithelial defense, thereby contributing to the active regulation of the local microenvironment as well as to epithelial homeostasis or, when dysregulated, cellular stress and damage [110,111]. In immune cells, mitochondrial metabolism can influence how microbial signals are translated into inflammatory and antimicrobial responses [112,113,114,115]. Mitochondria may therefore act as intracellular translators of microbial signals, contributing to qualitatively different cellular outcomes rather than a uniform response to microbial exposure. Within the proposed vaginal microbiome–mitochondria axis, the translation of microbial signals by mitochondria provides the conceptual link between the molecular signals discussed above and the distinct epithelial and immune phenotypes observed across vaginal conditions.

Vulvovaginal candidiasis provides a direct model in which mitochondrial responses participate in epithelial host defense. During *Candida* infection, vaginal epithelial cells undergo early mitochondrial alterations, including changes in mitochondrial membrane potential and morphology and the release of mtROS and mitochondrial DNA [116]. These mitochondrial changes occur before overt epithelial damage, indicating that they are not simply a consequence of cellular injury [116]. Mitochondrial DNA released into the cytosol induces type I interferon signaling, which increases epithelial resistance to *Candida*-induced damage, although the pathway linking mtDNA release to type I interferon activation in vaginal epithelial cells has not been fully defined [116]. In parallel, mtROS contribute to epithelial control of *C. albicans* growth, as pharmacological scavenging of mtROS increases fungal burden during epithelial infection without directly promoting fungal growth [12]. At later stages of the interaction, *C. albicans* virulence mechanisms can shift the epithelial response toward cellular injury, with candidalysin contributing to mitochondrial activation, mtROS production, and epithelial damage [12,101]. *Candida* therefore illustrates a dynamic role of mitochondria in vaginal epithelial cells, in which early mitochondrial signaling contributes to epithelial defense, whereas more pronounced mitochondrial perturbation accompanies the damage-driven response that develops as infection progresses.

The role of mitochondria as translators of microbial signals is not restricted to epithelial cells but extends to immune cells, in which mitochondrial function contributes to the regulation of antimicrobial and inflammatory responses. Neutrophils provide a particularly relevant example. Although mature neutrophils rely predominantly on glycolysis for ATP production, their mitochondria remain functionally important in processes beyond cellular bioenergetics [117,118]. Rather than serving primarily as a source of cellular energy, mitochondria participate in the regulation of several steps of the neutrophil response to microbial stimulation. Mitochondrial ATP can contribute to purinergic signaling involved in cell polarization and chemotaxis [119], while mtROS participate in redox signaling and in the activation of antimicrobial effector mechanisms [117,118]. Mitochondrial function has also been implicated in neutrophil extracellular trap (NET) formation, providing a further mechanism through which mitochondrial activity can influence the interaction between neutrophils and microorganisms. Thus, even in a predominantly glycolytic immune cell, mitochondria can contribute to the transition from microbial sensing to cell migration and antimicrobial activity.

Among the mitochondrial mechanisms contributing to neutrophil antimicrobial activity, mtROS provide one of the clearest functional links between mitochondrial activity and microbial killing. Although the oxidative burst of neutrophils is primarily associated with NOX2-derived ROS, mitochondria represent an additional source of ROS that can participate directly in antimicrobial responses. In human neutrophils exposed to *Staphylococcus* aureus, inhibition of mitochondrial complex III reduces mtROS production, NET formation, and bacterial killing, while selective scavenging of mtROS similarly impairs bactericidal activity, demonstrating that mitochondrial ROS can make a functional contribution to microbial clearance [120]. Mitochondrial involvement in NET formation provides a further connection between cellular metabolism and antimicrobial activity. NETs consist of extracellular DNA associated with granular and antimicrobial proteins that can immobilize microorganisms and concentrate antimicrobial effectors in their proximity [121]. Their formation, however, is mechanistically heterogeneous, and mitochondrial involvement varies according to the activating stimulus [122]. In response to *S. aureus*, mitochondrial complex III and mtROS contribute to NET formation [120], whereas GM-CSF priming followed by LPS or C5a stimulation can induce ROS-dependent release of mitochondrial DNA (mtDNA) into NETs by viable neutrophils [123]. By contrast, other pathways, such as PMA-induced NET formation, rely predominantly on NOX2-derived ROS and glycolytic metabolism rather than mitochondrial oxidative metabolism [122]. Mitochondria can therefore support neutrophil antimicrobial activity through distinct and stimulus-dependent mechanisms, including mtROS production and, under specific conditions, mitochondrial participation in NET formation.

The activation of neutrophil antimicrobial mechanisms does not, however, ensure effective microbial clearance. Vulvovaginal candidiasis provides a particularly clear example of this dissociation. Symptomatic *C. albicans* infection is characterized by extensive neutrophil recruitment, yet the infiltrating neutrophils may fail to reduce the vaginal fungal burden and instead contribute to local immunopathology [124]. This apparent paradox can result from modulation of neutrophil function by the vaginal microenvironment. Vaginal heparan sulfate can bind Mac-1 on neutrophils and interfere with its interaction with the fungal ligand Pra1, thereby impairing recognition and killing of *C. albicans* [13]. Experimental degradation of heparan sulfate restores neutrophil antifungal activity [13], and recent studies using vaginal samples from women with symptomatic VVC have confirmed an association between increased heparan sulfate levels and impaired neutrophil-mediated fungal killing [14]. In another study, significantly higher levels of cell-associated perinuclear anti-neutrophil cytoplasmic antibodies (pANCA) were detected in the cellular fraction of vaginal samples from women with VVC than in healthy *Candida*-colonized women [125]. In vitro, anti-MPO antibodies markedly impaired neutrophil candidacidal activity while stimulating ROS production, an effect further enhanced by exposure to *C. albicans* [125]. These findings suggest that pANCA may contribute to neutrophil dysfunction in VVC by impairing candidacidal activity while promoting an exaggerated ROS response that could contribute to local tissue damage.

Thus, recruitment and activation of neutrophils cannot be equated with an effective antimicrobial response: the functional outcome also depends on how neutrophil effector mechanisms are modulated by the local microenvironment.

The magnitude of neutrophil recruitment alone does not define the functional outcome of the vaginal immune response. This is evident across vaginal conditions and even within the same condition. In VVC, extensive neutrophil recruitment can coexist with ineffective fungal clearance, as discussed above [124]. By contrast, AV is characterized by pronounced inflammatory activation in a distinct microbial environment, whereas BV typically shows a comparatively limited neutrophilic response despite substantial microbial and metabolic disruption [7,8]. The local microenvironment in which neutrophils operate is itself partly shaped by microbe–epithelial interactions. In vaginal epithelial cells, *C. albicans* induces transcriptional changes involving pathways related to heparan sulfate biosynthesis and modification, while epithelial damage and repair may further influence its extracellular availability [13]. Thus, microbial activity can indirectly modify immune-cell function by altering epithelial responses and, consequently, the local environment. Conversely, experimental models show that neutrophil recruitment can result in effective fungal clearance when this inhibitory effect is absent [13]. Moreover, the magnitude of neutrophil recruitment is not determined by fungal burden alone but can vary according to specific fungal signals and virulence properties; candidalysin, for example, promotes epithelial inflammatory signaling and neutrophil recruitment without significantly affecting vaginal colonization [10]. Aerobic vaginitis provides a different inflammatory setting, characterized by increased numbers of leukocytes, including toxic leukocytes, although their presence indicates inflammatory activation rather than demonstrating effective bacterial clearance [7,8]. Bacterial vaginosis, in contrast, is generally associated with a less pronounced neutrophilic response despite profound changes in microbial composition and metabolic activity [126,127]. Recruitment, activation, and antimicrobial efficacy should therefore be considered distinct dimensions of the neutrophil response rather than interchangeable measures of host defense. The resulting inflammatory phenotype reflects not only the extent of neutrophil recruitment, but also the microbial signals that activate these cells, the local microenvironment in which they operate, and the effector programs they engage.

Within the proposed vaginal microbiome–mitochondria axis, neutrophils therefore provide an example of how mitochondrial signaling may contribute to translating microbial stimulation into a functional host response. Microbial and microenvironmental signals can shape neutrophil activation, while mitochondrial pathways, particularly mtROS-dependent signaling and, under specific conditions, NET formation, may influence the resulting antimicrobial or inflammatory response. The functional outcome cannot therefore be inferred from neutrophil recruitment alone, but depends on how microbial signals, the local vaginal environment, and intracellular mitochondrial responses interact to determine effective microbial control or potentially maladaptive inflammation.

Taken together, these observations indicate that the vaginal host response emerges from a complex network of interactions between microbial signals, epithelial cells, the local microenvironment, and immune cells. Microbial signals can act directly on host cells or indirectly by modifying epithelial responses and the environment in which immune cells operate. The resulting cellular response therefore depends not only on the nature of the extracellular stimulus, but also on how that stimulus is sensed and translated within the responding cell. Effective mucosal defense therefore requires not only microbial recognition and antimicrobial activity, but also the coordinated regulation of inflammation, epithelial integrity, and tissue recovery.

## 7. From Mitochondrial Eustress to Failure of Resolution

The biological consequences of mitochondrial responses to microbial signals depend on their intensity, duration, localization, and capacity for resolution [22,112,128]. Mitochondrial activation is not inherently detrimental: appropriately regulated changes in bioenergetics, redox signaling, and mitochondrial dynamics can support cellular adaptation and antimicrobial defense [112,128]. The same pathways, however, may become maladaptive when activation is excessive, prolonged, or inadequately resolved [128]. Within the proposed vaginal microbiome–mitochondria axis, the relevant distinction is therefore not simply between mitochondrial activation and inactivity, but between adaptive mitochondrial responses that support host defense and recovery and dysfunctional responses that contribute to inflammation, cellular damage, or impaired microbial control.

The functional consequences of this mitochondrial response depend on how it is regulated in space, time, and biological context. This is particularly evident for mtROS. Mitochondrial ROS can participate in intracellular redox signaling [129], but they can also contribute directly to antimicrobial effector mechanisms, including the oxidative response of neutrophils [120,130]. Thus, oxidative eustress should not be equated simply with low ROS production: even a substantial oxidative response may remain physiologically functional when it is appropriately generated, compartmentalized, and terminated [129,131]. Oxidative distress arises instead when redox regulation is disrupted and ROS production causes unintended molecular and cellular damage or sustains pathological inflammatory signaling [130,131]. In host–microbe interactions, the distinction between mitochondrial eustress and distress therefore depends less on the absolute amount of mtROS than on whether the oxidative response remains functionally directed and effectively resolved. No single quantitative threshold currently distinguishes adaptive mitochondrial responses from pathological dysfunction across cell types and biological contexts. Experimentally, this distinction therefore requires integrated assessment of the magnitude and temporal resolution of mitochondrial perturbation together with functional outcomes. Relevant measurable parameters include mitochondrial respiration and ATP production, membrane potential, mtROS generation and redox balance, mitochondrial dynamics and quality-control responses, and evidence of oxidative damage. An adaptive response would be expected to remain reversible and compatible with preserved or restored cellular function, whereas persistent bioenergetic impairment, dysregulated redox signaling, defective quality control, or molecular damage would support mitochondrial dysfunction. These criteria remain to be validated specifically in vaginal epithelial and immune cells.

When mitochondrial homeostasis is disrupted, mitochondrial signaling may become dysregulated and alter the host response in different directions [112,132]. Signals that normally contribute to a regulated response may become excessive or persistent, amplifying inflammation and tissue damage [130], or mitochondrial dysfunction may impair the cellular functions required for effective antimicrobial defense [112,133]. Mitochondrial distress may therefore result either in an exaggerated and poorly resolved response or in an inadequate response to the microbial stimulus, potentially favoring microbial persistence.

Mitochondrial distress does not necessarily result in persistent dysfunction. Cells can activate mitochondrial quality-control mechanisms that limit damage and restore mitochondrial homeostasis [134,135]. Damaged components can be repaired or removed, dysfunctional mitochondria can be selectively eliminated through mitophagy [135,136], and the mitochondrial network can be restored through biogenesis and dynamic remodeling [134,137]. The outcome of mitochondrial stress therefore depends not only on the initial perturbation, but also on the ability of the cell to recover from it. Effective mitochondrial recovery can terminate stress signaling and support restoration of cellular homeostasis, whereas incomplete recovery may allow mitochondrial dysfunction and its effects on the host response to persist [135,136].

Mitochondrial recovery may also depend on effective control of the microbial stimulus. Persistent microbial activity can repeatedly perturb mitochondrial function [138], while incomplete mitochondrial recovery may impair the host response required for microbial control [135,136]. These processes could therefore reinforce each other, preventing complete resolution of the host response.

Incomplete mitochondrial recovery may influence how host cells respond to subsequent microbial stimuli [139]. Residual mitochondrial dysfunction may alter their metabolic, redox, or immune capacity, potentially affecting the effectiveness of the response to a new microbial challenge [139,140].

## 8. Recurrence as a Failure of Ecosystem–Host Reset

Resolution of vaginal infection or dysbiosis requires restoration of both the microbial ecosystem and the host response. Because these components are interdependent, incomplete recovery of either may prevent restoration of a stable vaginal environment [141,142,143]. Recurrence may therefore reflect a failure of the microbial ecosystem–host to achieve a complete and stable reset after the initial episode.

Clinical resolution does not necessarily imply biological restoration of the vaginal ecosystem. Treatment may reduce microbial burden and symptoms while leaving residual microbial communities, biofilm, or an incompletely restored host environment that favors renewed dysbiosis or infection [141,142]. Moreover, successful restoration should not be understood simply as the return of the vaginal microbiota to a predefined taxonomic composition, but as the re-establishment of a stable functional relationship between the microbial community and the host. Recurrence may therefore arise from a biologically altered state in which this relationship has not been fully restored, rather than from a completely recovered ecosystem undergoing a new microbial perturbation.

Within this framework, mitochondrial recovery may represent a potentially critical determinant of the microbial ecosystem–host reset. Persistent mitochondrial dysfunction could alter epithelial and immune responses after the initial episode, potentially impairing the capacity of the vaginal mucosa to restore and maintain a local environment compatible with microbial equilibrium [112,144]. Conversely, persistent dysbiosis or infection may continue to challenge mitochondrial homeostasis [136], creating the potential for reciprocal failure of microbial and host recovery.

Importantly, mitochondrial dysfunction may outlast the microbial stimulus that initially induced it [145]. Evidence from other infectious models indicates that mitochondrial alterations can persist beyond the acute phase of infection, providing a potential mechanism through which a transient microbial perturbation may produce longer-lasting changes in host-cell function [139,145]. Whether a similar mitochondrial legacy persists after vaginal infection or dysbiosis remains hypothetical. No longitudinal clinical or experimental studies have yet established whether mitochondrial dysfunction persists after resolution of vaginal infection or dysbiosis, or whether such persistence is associated with subsequent recurrence. This therefore represents a key knowledge gap and a directly testable component of the proposed framework.

One possible mechanism involves the ability of persistent mitochondrial dysfunction to modify the mucosal environment itself. Impaired oxidative metabolism may reduce the energetic and metabolic capacity required to maintain epithelial integrity, regeneration, and antimicrobial functions [24,146], while dysregulated mtROS production can promote oxidative damage and sustain inflammatory signaling [25,147]. Mitochondrial damage may also result in the release of mtDNA and other mitochondrial danger signals, further activating innate immune pathways [19]. Importantly, evidence from the intestinal mucosa indicates that altered epithelial mitochondrial metabolism can modify the extracellular environment and thereby influence microbial ecology, for example by changing oxygen availability, antimicrobial peptide production, and epithelial barrier function [24,25]. If analogous mechanisms operate in the vagina, incomplete mitochondrial recovery could alter both epithelial and immune control of the local environment, potentially creating conditions that favor persistence or re-emergence of dysbiosis. Persistent microbial activity could in turn provide renewed metabolic, inflammatory, or toxic signals to mitochondria, establishing a self-reinforcing cycle between mitochondrial dysfunction and failure of ecosystem–host recovery.

If mitochondrial dysfunction contributes to failure of microbial ecosystem–host reset, microbial eradication alone may not restore the biological conditions required for durable resolution. This possibility raises the question of whether treatment should extend beyond control of the microbial trigger to support restoration of host and microbial ecosystem function.

**Figure 2:**
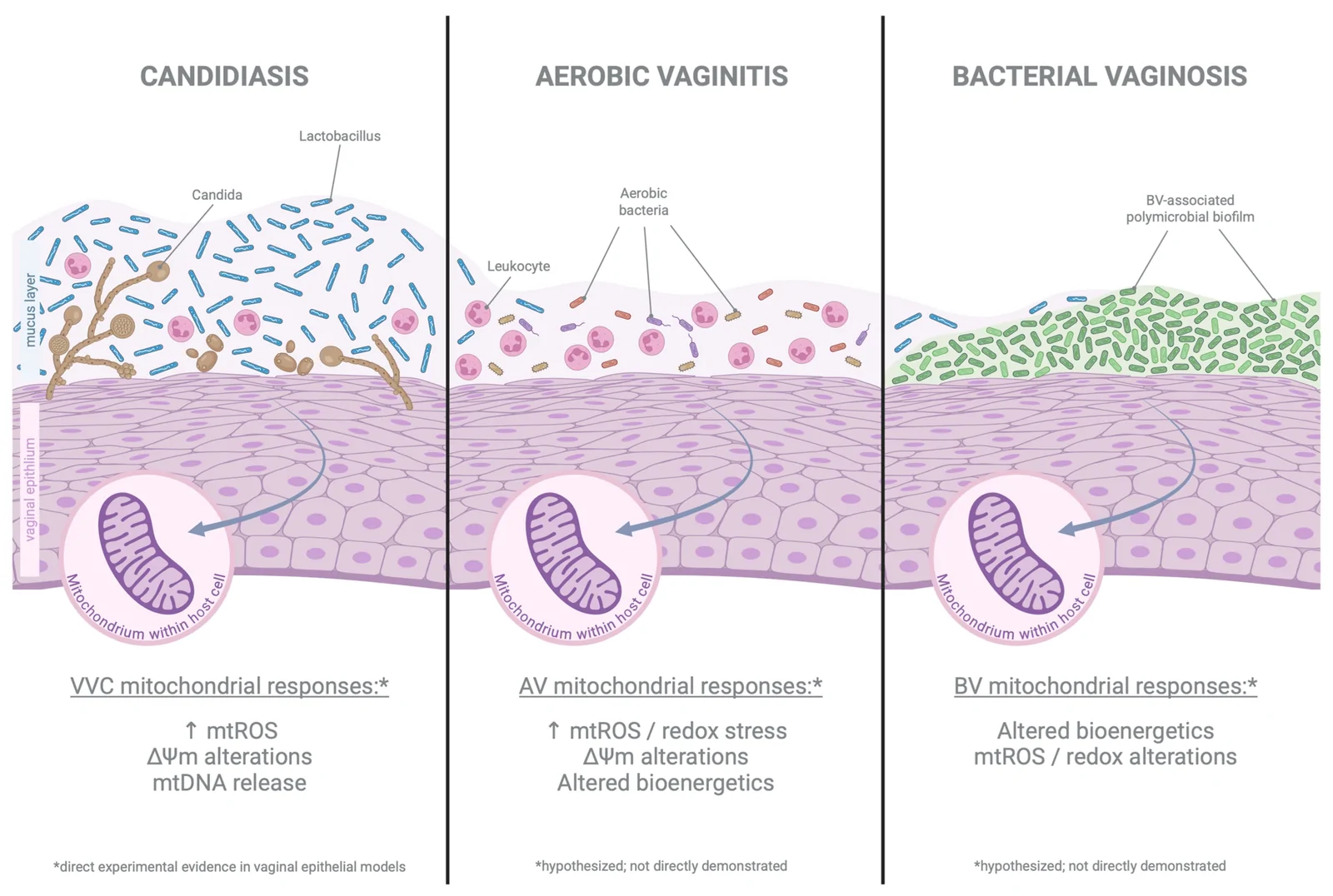
Distinct vaginal conditions and their proposed mitochondrial responses. Bacterial vaginosis (BV), aerobic vaginitis (AV), and vulvovaginal candidiasis (VVC) are characterized by distinct microbial and mucosal inflammatory profiles, which may expose host cells to different metabolic, redox, and virulence-associated signals. In VVC, increased mitochondrial reactive oxygen species (mtROS), alterations in mitochondrial membrane potential (ΔΨm), and mitochondrial DNA (mtDNA) release have been directly demonstrated in experimental vaginal epithelial models. By contrast, the mitochondrial responses depicted for AV and BV are hypotheses derived from established features of their microbial and metabolic environments and from mechanistic evidence showing that relevant microbial products, metabolites, or virulence factors can modulate the indicated mitochondrial pathways in other experimental systems. Thus, the AV and BV profiles represent biologically supported extrapolations rather than mitochondrial phenotypes demonstrated in vaginal tissue or clinical disease. The figure illustrates testable predictions of the vaginal microbiome–mitochondria axis and does not imply that the proposed mitochondrial alterations are specific to, or invariably present in, each condition. Created in https://BioRender.com.

Within this framework, BV, AV, and VVC may represent distinct examples of how different microbial ecosystems and host responses could engage the microbiome–mitochondria axis. In BV, profound ecological and metabolic changes occur in the presence of a relatively limited neutrophilic response; AV is characterized by marked inflammatory activation associated with a different microbial environment; and VVC illustrates how an intense neutrophilic response may coexist with ineffective microbial clearance and epithelial damage. Whether these distinct biological phenotypes are associated with correspondingly distinct patterns of mitochondrial adaptation or dysfunction remains unknown. Their comparison therefore provides a clinically relevant setting in which to test whether mitochondrial responses contribute to the different outcomes of vaginal microbe–host interactions (Figure 2).

## 9. Targeting the Axis: From Antimicrobial Treatment to Metabolic Restoration

Conventional treatment of vaginal infections and dysbiosis is primarily directed at controlling the microorganisms associated with the disease. This remains essential, but microbial suppression does not necessarily ensure restoration of the microbial ecosystem–host relationship disrupted during the episode [141]. Antimicrobial treatment may itself modify microbial community structure and function and, depending on the agent and biological context, may also influence host-cell metabolism and mitochondrial function [148]. Treatment of recurrent vaginal conditions may therefore need to be considered not only in terms of antimicrobial efficacy, but also in terms of its capacity to support recovery of mucosal homeostasis after microbial control.

The bacterial ancestry of mitochondria provides a biological basis for some antibiotics to interfere with mitochondrial function [148]. Depending on the drug and cellular context, antibiotics can affect mitochondrial protein synthesis, respiratory chain activity, membrane potential, ATP production, and ROS generation [148,149]. These effects are highly heterogeneous and cannot be considered a general property of antimicrobial therapy; importantly, several observations derive from non-vaginal cells, experimental models, or drug exposures that may not reflect concentrations reached in the vaginal mucosa [148,149]. Nevertheless, mitochondrial effects have also been reported for agents relevant to the treatment of vaginal conditions, including metronidazole and clindamycin, although their nature and magnitude vary across experimental models [148,150].

The therapeutic implication is therefore not that antimicrobial treatment should be avoided, but that microbial control may represent only the first step toward biological recovery. Once the microbial disturbance has been controlled, restoration of the vaginal ecosystem requires re-establishment of conditions that support a stable microbial community and recovery of epithelial and immune homeostasis [151,152]. Within the proposed microbiome–mitochondria axis, mitochondrial recovery may contribute to this second phase by restoring the metabolic and redox capacity required for normal host-cell function [138].

Metabolic restoration should not be understood as the nonspecific stimulation of mitochondrial activity, but as recovery of mitochondrial fitness appropriate to the functional needs of epithelial and immune cells. Depending on the underlying disturbance, this may involve restoration of oxidative metabolism and redox balance, removal of damaged mitochondria through mitophagy, and renewal of the mitochondrial pool through biogenesis [134,153,154]. Within the proposed vaginal microbiome–mitochondria axis, the therapeutic objective would therefore be restoration of mitochondrial homeostasis rather than modulation of any single mitochondrial pathway.

Such interventions would need to preserve physiological and adaptive mitochondrial functions rather than indiscriminately suppress mitochondrial activity. This is particularly relevant to mtROS, because mitochondrial oxidant signaling can contribute to antimicrobial defense, and the pharmacological reduction in mtROS has been associated with impaired microbial control, including increased *C. albicans* burden in a vaginal epithelial model [12,120].

Restoration of mitochondrial homeostasis could potentially be approached at several levels. Mitochondrial oxidative damage may be limited by targeting excessive mtROS, while damaged mitochondria can be removed through mitophagy and the functional mitochondrial pool restored through biogenesis [112,138]. Experimental infection, inflammatory, and mucosal models further indicate that mitochondrial redox regulation, quality control, bioenergetics, and metabolic signaling can be pharmacologically modulated, in some settings improving epithelial or immune function [155,156,157]. Table 2 presents selected illustrative examples chosen for their mechanistic relevance to different components of mitochondrial homeostasis and should not be considered an exhaustive survey of mitochondria-targeted compounds. These observations provide proof of principle that mitochondrial recovery is pharmacologically modifiable, although whether such approaches can promote recovery of the vaginal ecosystem remains to be established.

The therapeutic potential of the microbiome–mitochondria axis extends beyond direct pharmacological targeting of mitochondria. Because mitochondrial function is influenced by the surrounding microbial and metabolic environment, restoration of mitochondrial homeostasis could potentially be pursued both directly, through mitochondria-targeted interventions, and indirectly, by modifying the microbial signals to which host cells are exposed. This distinction broadens the therapeutic framework from mitochondrial pharmacology to interventions aimed at restoring functional communication between the vaginal ecosystem and the host.

Direct mitochondrial targeting could therefore act at different levels of mitochondrial homeostasis, including redox regulation, quality control, bioenergetics, biogenesis, and mitochondrial dynamics. Importantly, these strategies do not converge on a single mitochondrial response but may restore different components of mitochondrial fitness depending on the underlying dysfunction. At present, however, it remains unclear which mitochondrial alterations represent the most relevant therapeutic targets in different infectious or inflammatory settings. This uncertainty is even greater in the vaginal environment, where mitochondrial dysfunction has not yet been systematically characterized and the therapeutic potential of direct mitochondrial targeting remains unexplored.

Indirectly, mitochondrial homeostasis could be promoted by modifying the microbial environment responsible for generating mitochondrial stress. Restoration of a more favorable vaginal ecosystem could reduce exposure to microbial metabolites, toxins, and inflammatory signals capable of perturbing mitochondrial function, while restoring signals that support normal host-cell metabolism. In this perspective, interventions directed at the vaginal microbiota could have downstream mitochondrial effects even without directly targeting mitochondria.

Strategies aimed at restoring *Lactobacillus* dominance can reshape the vaginal ecosystem and its metabolic output, thereby modifying the signals reaching host mitochondria. Lactic acid and H_2_O_2_ produced by vaginal lactobacilli have been shown to modulate mitochondrial membrane potential and functional activity in human neutrophils [168], while metabolites released by vaginal *Lactobacillus* strains similarly affect mitochondrial potential in epithelial cells [169]. Beyond these physiological interactions, microbial-derived products can themselves be exploited experimentally to modulate mitochondrial and host-cell function. Table 3 presents selected illustrative examples chosen to represent different microbial-derived interventions and mitochondrial targets, rather than an exhaustive survey of microbial products that can modulate mitochondrial activity. This possibility extends indirect targeting of the microbiome–mitochondria axis from restoration of microbial composition to the use of microbial-derived interventions capable of promoting mitochondrial, epithelial, or immune recovery.

The examples summarized in Table 3 illustrate that microbial-derived interventions can influence mitochondrial homeostasis through distinct mechanisms, ranging from modulation of mitochondrial redox and bioenergetics to mitophagy, biogenesis, and dynamics. Importantly, these effects have been observed not only with individual microbial metabolites but also with more complex microbial preparations, including supernatants and lysates. Although most evidence still derives from experimental models outside the vaginal environment, these findings provide proof of principle that the microbial side of the microbiome–mitochondria axis can itself be therapeutically exploited to influence host mitochondrial function.

Direct and indirect targeting should not be regarded as alternative therapeutic strategies. Their potential value lies in acting on complementary components of the same biological system: controlling the microbial disturbance, restoring a favorable metabolic environment, and allowing recovery of mitochondrial, epithelial, and immune homeostasis. Rather than simply adding mitochondrial modulators to existing antimicrobial treatment, this framework therefore suggests a shift toward interventions designed to promote coordinated microbial ecosystem–host recovery. Translating this concept into therapy will first require defining which mitochondrial alterations occur in specific vaginal conditions, whether they persist after microbial control, and which of them are reversible or therapeutically actionable.

## 10. Conclusions and Testable Hypotheses

The evidence reviewed here supports a conceptual framework in which mitochondria may represent a functional interface between the vaginal microbiota and the host. Microbial metabolites and virulence factors can influence mitochondrial bioenergetics, redox signaling, dynamics, and quality control pathways, while mitochondrial responses can in turn shape epithelial integrity, antimicrobial activity, inflammation, and immune function. Most of these mechanistic connections have not yet been demonstrated directly in the vaginal environment, and the vaginal microbiome–mitochondria axis should therefore be regarded as a testable biological model rather than an established pathogenic pathway.

These questions can be organized into a stepwise experimental agenda, progressing from demonstration of vaginal mitochondrial phenotypes to mechanistic testing of causality, longitudinal assessment of mitochondrial recovery, and ultimately evaluation of therapeutic modulation.

1. Are distinct vaginal conditions associated with distinct mitochondrial phenotypes?

Do BV, AV, VVC, and other vaginal conditions, characterized by different microbial, metabolic, and inflammatory profiles, differ in mitochondrial respiration, membrane potential, mtROS generation, dynamics, or quality-control pathways in vaginal epithelial and immune cells? Establishing such associations would provide direct evidence that mitochondrial function varies across biologically distinct states of the vaginal ecosystem.

2. Are mitochondria active mediators of host–microbe interactions rather than merely targets of microbial activity?

Can microbial metabolites and virulence factors characteristic of specific vaginal conditions reproduce mitochondrial alterations in vaginal epithelial or immune cells, and does experimental manipulation of mitochondrial redox signaling, bioenergetics, dynamics, mitophagy, or biogenesis modify epithelial barrier function, antimicrobial activity, or inflammatory and immune responses to microbial challenge? These experiments could establish both the directionality and functional relevance of microbial–mitochondrial interactions.

3. Does incomplete mitochondrial recovery contribute to persistence or recurrence?

Does microbiological or clinical resolution precede complete restoration of mitochondrial, epithelial, and immune homeostasis, and is residual mitochondrial dysfunction associated with subsequent recurrence? Longitudinal studies could determine whether mitochondrial recovery follows a different temporal trajectory from microbial clearance and whether restoration of mitochondrial homeostasis, either directly or through modification of the microbial environment, favors durable microbial ecosystem–host reset.

4. How do antimicrobial treatments interact with mitochondrial recovery?

Do antibiotics and other antimicrobial treatments restore mitochondrial homeostasis primarily by removing microbial triggers, or can their direct effects on host mitochondria modify, delay, or interfere with biological recovery? Comparing microbial clearance with mitochondrial function before, during, and after treatment could distinguish the consequences of pathogen or dysbiosis control from treatment-associated mitochondrial effects and determine whether therapeutic strategies should consider both microbial eradication and host metabolic recovery.

The central proposition of the vaginal microbiome–mitochondria axis is therefore not that mitochondria constitute another isolated mechanism in vaginal disease but that they may provide a functional link between changes in the microbial ecosystem and the capacity of the vaginal mucosa to respond, recover, and maintain local homeostasis. Determining whether this link exists in vivo, whether it differs across vaginal disorders, and whether it can be therapeutically modified could shift the study of recurrent vaginal disease from repeated microbial eradication toward understanding—and ultimately restoring—the biological conditions required for durable resolution.

## Figures and Tables

**Table 1:** Microbial Signals Potentially Linking the Vaginal Microbiome to Mitochondrial Function.

Molecule	Relevant Source	Potential Vaginal Relevance	Mitochondrial Effects	Evidence	Tissue/Cell Type	References
L-Lactate	*Lactobacillus* spp.	Homeostasis	↑ respiration; ↑ ATP; ↑ H_2_O_2_	In vitro	Alveolar type II epithelial cells; hepatocytes	[39,42,43]
D-Lactate	*Lactobacillus* spp.	Homeostasis	↑ ETC activity; ↑ respiration; ↑ ATP	In vitro	CD8+ T cells	[43,44]
Acetate (SCFA)	Anaerobic bacteria	BV	↑ respiration/OxPhos; ↑ ATP; ↑ SRC; ↑ mitochondrial density; altered ROS; altered fission	In vitro; in vivo	Pancreatic β cells; colon epithelial cells; renal tubular epithelial cells	[45,46,47,48,49,50]
Propionate (SCFA)	Anaerobic bacteria	BV	altered respiration/OxPhos; altered ETC activity; ↑ ATP; ↑ SRC; altered ΔΨm; altered fission/fusion; altered biogenesis	In vitro; ex vivo; in vivo	Adipocytes/adipose tissue; fibroblasts; hepatic cells; neuronal cells	[51,52,53]
Butyrate (SCFA)	Anaerobic bacteria	BV	altered respiration/OxPhos; altered ATP; ↑ SRC; altered ΔΨm; altered fission/fusion; ↑ mass; ↑ biogenesis signaling	In vitro; ex vivo; in vivo	Colonocytes/colonic epithelial cells; pancreatic β cells; lymphoblasts; glomerular endothelial cells; motor neuron-like cells	[45,54,55,56,57]
Succinate	Anaerobic bacteria	BV	↑ complex II/ETC activity; altered respiration/OxPhos; ↑ mtROS/RET; ↓ content; ↓ UPRmt; ↓ ATP	In vitro; ex vivo; in vivo	Macrophages; cardiac and neuronal tissue; *C. elegans*	[58,59,60]
Putrescine (BA)	Anaerobic bacteria	BV	altered respiration; ↑ SRC; ↑ ATP; uncoupling; ↑ cytochrome c release	In vitro	Intestinal epithelial cells; hepatic tissue; lymphoma cells	[61,62,63]
Cadaverine (BA)	*Enterobacteriaceae*; anaerobic bacteria	BV; AV	altered respiration/OxPhos; altered ΔΨm; ↓ oxidation; ↑ lipid peroxidation	In vitro; in vivo	Monocytes/macrophages; breast cancer cells; hepatic tissue	[64,65,66,67]
Tyramine (BA)	*Enterococcus* spp.; *Lactobacillus* spp.; anaerobic bacteria	BV; AV	↓ respiration; ↑ mtH_2_O_2_; ↑ mtDNA damage; ↓ ΔΨm; ↑ permeability transition/swelling	In vitro	Brain and hepatic tissue; myoblasts	[68,69,70,71]
Spermidine (BA)	*Dialister* spp.; *Megasphaera* spp.	BV	↑ respiration/OxPhos; ↑ ATP; ↑ SRC; ↑ ΔΨm; ↓ mtROS; ↑ mitophagy; altered number/morphology	In vitro; in vivo	Neuronal cells; macrophages; cardiac tissue	[72,73,74,75,76]
Spermine (BA)	*Lactobacillus* spp.; *Enterobacteriaceae*; other bacteria	BV; AV	↓ respiration; ↓ ATP; ↓ ΔΨm; ↑ mtROS; ↓ number; ↓ fusion; altered permeability transition; altered lipid peroxidation	In vitro; in vivo	CD8+ T cells; hepatic tissue	[77,78,79,80]
Agmatine (BA)	*Prevotella bivia*; other anaerobic bacteria	BV	altered respiration; altered ATP; altered ROS; altered ΔΨm; altered permeability transition	In vitro	Hepatic, renal and brain tissue	[81,82,83]
Trimethylamine	*Parvimonas micra*; *Finegoldia magna*; *Mobiluncus* spp.	BV	↓ ATP; ↑ ΔΨm; ↓ mtDNA copy number; altered gene expression; altered mtDNA methylation	In vitro	Intestinal epithelial cells; monocytes	[84,85]
Hydrogen peroxide	*Lactobacillus jensenii*; *L. crispatus*; *L. gasseri*	Homeostasis	altered respiration; ↓ ETC/TCA enzyme activity; ↑ mtDNA damage	In vitro	Cardiac tissue; retinal pigment epithelial cells; corneal epithelial cells	[86,87,88]
Cytotoxic necrotizing factor 1	*Escherichia coli*	AV	↑ ATP; ↑ ETC/OxPhos activity; ↑ elongation/networking; ↓ pathological mtROS	In vitro; in vivo	Intestinal crypt epithelial cells; skin fibroblasts; brain tissue	[89,90,91,92]
Hemolysin A	*Escherichia coli*	AV	↑ Ca^2+^; ↑ fragmentation; ↓ ΔΨm; ↓ ATP; ↑ cytochrome c release	In vitro	Sertoli cells; intestinal epithelial cells; brain microvascular endothelial cells; macrophages	[93,94,95]
Alpha-toxin	*Staphylococcus aureus*	AV	↑ Ca^2+^; ↓ ΔΨm; ↑ cytochrome c release	In vitro	T lymphocytes; airway epithelial cells	[96,97]
Panton–Valentine leukocidin	*Staphylococcus aureus*	AV	↓ ΔΨm; ↑ cytochrome c release; ↑ Smac/DIABLO release	In vitro	Neutrophils; hepatic tissue	[98]
Granadaene	*Streptococcus agalactiae*	AV	↓ ΔΨm; ↑ uncoupled respiration; ↓ ATP synthesis; ↑ cytochrome c release; ↓ Ca^2+^ uptake	In vitro	Cardiomyocytes; brain tissue	[99,100]
Candidalysin	*Candida albicans*	VVC	↑ mtROS; ↓ ΔΨm; ↓ ATP; ↑ cytochrome c release	In vitro	Vaginal epithelial cells; oral epithelial cells	[12,101]
Farnesol	*Candida albicans*	VVC	↓ respiration; ↓ SRC; ↓ ATP-linked respiration; ↓ ΔΨm; ↑ cytochrome c release	In vitro	Monocytes/dendritic cells; T-lymphoblastic cells	[102,103]
Indole-3-propionic acid	Anaerobic bacteria	BV	↑ respiration/OxPhos; ↑ ΔΨm; ↓ mtROS; altered cristae morphology	In vitro; in vivo	CD4+ T cells; cervical cancer cells; mouse embryonic fibroblasts	[104]
Indole-3-lactic acid	*Lactobacillus* spp.	Homeostasis	↑ complex IV activity; ↑ COX5B expression; ↓ ROS	In vitro	Hepatic cancer cells	[105]
Indole-3-aldehyde	*Lactobacillus* spp.	Homeostasis	↑ respiration; ↑ SRC; ↑ ΔΨm; ↓ mtROS	In vitro; in vivo	Endothelial cells	[106,107]
Indole	Anaerobic bacteria	BV	↓ respiration; ↓ SRC; ↓ ATP; uncoupling	In vitro; ex vivo	Intestinal epithelial cells; hepatic tissue	[108]
Hydrogen sulfide	Anaerobic bacteria	BV	altered respiration; altered complex IV activity; altered ATP; altered ΔΨm	In vitro; ex vivo	Colon epithelial cells	[109]

The tissue/cell type column specifies the experimental context from which each mitochondrial effect was derived. Unless a vaginal model is explicitly indicated, these findings should be interpreted as mechanistic evidence supporting biological plausibility rather than as demonstration of the same mitochondrial effect in the vaginal environment. “Altered” indicates significant changes reported in different directions across experimental models or conditions. Abbreviations: AV, aerobic vaginitis; BA, biogenic amine; BV, bacterial vaginosis; COX5B, cytochrome c oxidase subunit 5B; ΔΨm, mitochondrial membrane potential; ETC, electron transport chain; mtDNA, mitochondrial DNA; mtH_2_O_2_, mitochondrial hydrogen peroxide; OxPhos, oxidative phosphorylation; RET, reverse electron transport; ROS, reactive oxygen species; SCFA, short-chain fatty acid; SRC, spare respiratory capacity; TCA, tricarboxylic acid cycle; UPRmt, mitochondrial unfolded protein response; VVC, vulvovaginal candidiasis.

**Table 2:** Pharmacological Modulators of Mitochondrial Homeostasis.

Compound	Target	Functional Effect	Evidence	Model	Potential Relevance	References
MitoQ	mtROS; mtDNA damage	↓ oxidative stress; ↑ mitochondrial and barrier function	In vitro; ex vivo; in vivo	Intestinal epithelium	Mitochondrial and epithelial protection	[111,158]
MitoTEMPO	mtROS	↓ oxidative stress; ↓ mtROS-dependent killing	In vitro; ex vivo; in vivo	Intestinal epithelial cells; neutrophils	Mitochondrial and epithelial protection	[111,120]
NR/NMN	NAD^+^; SIRT1/3–PGC-1α	↑ mitochondrial function; ↑ barrier and antimicrobial function	In vitro; ex vivo; in vivo	Intestinal epithelial cells; neutrophils; macrophages	Mitochondrial, epithelial, and immune recovery	[159,160]
Metformin	AMPK; complex I	↑ mtROS; ↑ phagosome maturation; ↓ bacterial load	In vitro; ex vivo; in vivo	Human and murine macrophages	Mitochondrial recovery; improved host defense	[161]
Elamipretide	Cardiolipin; inner mitochondrial membrane	↑ cristae integrity; ↑ ATP recovery	In vitro; ex vivo; in vivo; human/clinical	Renal epithelial/endothelial cells; skeletal muscle; human myocardium	Mitochondrial structural and bioenergetic recovery	[162,163,164]
ZLN005	PGC-1α–SIRT3	↑ mitochondrial repair; ↑ barrier function; ↓ bacterial load	In vitro; in vivo	Bronchial epithelial cells; phagocytes; murine infection	Mitochondrial, epithelial, and immune recovery	[156,165]
P110	Drp1–Fis1	↓ pathological fission; ↑ mitochondrial function; ↑ immune response	In vitro; in vivo	Intestinal epithelial cells; macrophages; murine sepsis	Mitochondrial, epithelial, and immune recovery	[166,167]

Abbreviations: AMPK, AMP-activated protein kinase; ATP, adenosine triphosphate; Drp1, dynamin-related protein 1; Fis1, mitochondrial fission 1 protein; mtDNA, mitochondrial DNA; mtROS, mitochondrial reactive oxygen species; NAD^+^, nicotinamide adenine dinucleotide; NMN, nicotinamide mononucleotide; NR, nicotinamide riboside; PGC-1α, peroxisome proliferator-activated receptor gamma coactivator 1-alpha; SIRT1/3, sirtuin 1/3. Potential relevance refers to the functional outcome that could theoretically support recovery of the vaginal ecosystem–host relationship and does not imply demonstrated therapeutic efficacy in vaginal infection or dysbiosis. Evidence categories refer to the experimental settings represented by the cited studies.

**Table 3:** Microbial-Derived Modulators of Mitochondrial Homeostasis.

Compound	Source	Target	Functional Effect	Evidence	Model	Potential Relevance	References
*Lactobacillus* supernatant	Vaginal lactobacilli	ΔΨm	ΔΨm modulation; metabolic activity; oxidative-stress response	In vitro	Cervical epithelial cells	Mitochondrial and epithelial modulation	[169]
*C. acnes* lysate	*C. acnes*	mtROS	↑ mtROS; ↓ cell damage; ↑ antimicrobial activity	In vitro	Vaginal epithelial cells; macrophages	Epithelial protection; improved host defense	[170]
Urolithin A	*G. urolithinfaciens*; *E. bolteae*; other gut microbiota taxa	Mitophagy	↑ mitophagy; ↓ mitochondrial dysfunction	In vitro; in vivo; human/clinical	Macrophages; intestinal epithelium	Mitochondrial and epithelial recovery	[171,172,173]
2,3-DHBA	*Enterobacteriaceae*	Mitochondrial biogenesis; dynamics; redox	↑ biogenesis; ↑ fusion; ↓ oxidative stress	In vivo	Intestinal mucosa	Mitochondrial and epithelial recovery	[174]
10-HSA	*L. plantarum*	PPARα; mitochondrial bioenergetics	↑ respiration; ↑ ATP-linked respiration; ↓ ROS	In vitro; ex vivo; in vivo	Intestinal epithelial cells; intestinal mucosa	Mitochondrial and epithelial recovery	[175]

Abbreviations: 2,3-DHBA, 2,3-dihydroxybenzoic acid; 10-HSA, 10-hydroxystearic acid; ATP, adenosine triphosphate; ΔΨm, mitochondrial membrane potential; mtROS, mitochondrial reactive oxygen species; PPARα, peroxisome proliferator-activated receptor alpha; ROS, reactive oxygen species. Potential relevance refers to the functional outcome that could theoretically support recovery of the vaginal ecosystem–host relationship and does not imply demonstrated therapeutic efficacy in vaginal infection or dysbiosis. Evidence categories refer to the experimental settings represented by the cited studies.

## Data Availability

No new data were created or analyzed in this study. Data sharing is not applicable to this article.

## Abbreviations

The following abbreviations are used in this manuscript:

AV	aerobic vaginitis
BV	bacterial vaginosis
COX5B	cytochrome c oxidase subunit 5B
ΔΨm	mitochondrial membrane potential
ETC	electron transport chain
IL-1RA	IL-1 receptor antagonist
LPS	Lipopolysaccharide
mtDNA	mitochondrial DNA
mtH_2_O_2_	mitochondrial hydrogen peroxide
mtROS	mitochondrial reactive oxygen species
NET	neutrophil extracellular trap
OxPhos	oxidative phosphorylation
pANCA	perinuclear anti-neutrophil cytoplasmic antibodies
RET	reverse electron transport;
ROS	reactive oxygen species
SCFA	short-chain fatty acid
SRC	spare respiratory capacity
TCA	tricarboxylic acid cycle
UPRmt	mitochondrial unfolded protein response
VVC	vulvovaginal candidiasis
